# The influence of inherited plumage colour morph on morphometric traits and breeding investment in zebra finches (*Taeniopygia guttata*)

**DOI:** 10.1371/journal.pone.0188582

**Published:** 2017-11-30

**Authors:** E. Tobias Krause, Oliver Krüger, Joseph I. Hoffman

**Affiliations:** 1 Department of Animal Behaviour, Bielefeld University, Bielefeld, Germany; 2 Institute of Animal Welfare and Animal Husbandry, Friedrich-Loeffler-Institut, Celle, Germany; Universite de Lausanne, SWITZERLAND

## Abstract

Melanin-based plumage polymorphism occurs in many wild bird populations and has been linked to fitness variation in several species. These fitness differences often arise as a consequence of variation in traits such as behaviour, immune responsiveness, body size and reproductive investment. However, few studies have controlled for genetic differences between colour morphs that could potentially generate artefactual associations between plumage colouration and trait variation. Here, we used zebra finches (*Taeniopygia guttata*) as a model system in order to evaluate whether life-history traits such as adult body condition and reproductive investment could be influenced by plumage morph. To maximise any potential differences, we selected wild-type and white plumage morphs, which differ maximally in their extent of melanisation, while using a controlled three-generation breeding design to homogenise the genetic background. We found that F_2_ adults with white plumage colouration were on average lighter and had poorer body condition than wild-type F_2_ birds. However, they appeared to compensate for this by reproducing earlier and producing heavier eggs relative to their own body mass. Our study thus reveals differences in morphological and life history traits that could be relevant to fitness variation, although further studies will be required to evaluate fitness effects under natural conditions as well as to characterise any potential fitness costs of compensatory strategies in white zebra finches.

## Introduction

Intraspecific variation in individual colouration is widespread across the animal kingdom [1,2,3]. This naturally occurring variation reflects underlying differences in the extent and pattern of pigment deposition, which in turn usually have a genetic basis. For example, the melanocortin-1 receptor (*MC1R*), which plays a central role in the deposition of black to brown eumelanin and the yellow to reddish-brown phaeomelanin, has been associated with plumage polymorphism in multiple bird species including, for example, bananaquits, snow geese, arctic skuas, red-footed boobies and chickens [1–4] as well as with hair colouration in humans and other mammals [5–7].

Despite many of the genes involved in pigmentation having no known pleiotropic effects, colour morphs of several species show significant differences in fitness-relevant life history traits. Notable exceptions are the melanocortin receptor genes which are known, in conjunction with the pro-opiomelanocortin (POMC) gene, to influence not only plumage pigmentation but also behaviour, immunity and stress responses [8]. For example, in barn and tawny owls, plumage colouration has been linked to fitness via differences in immune responsiveness [9–12]. Similarly, in common buzzards, three colour morphs termed 'light', 'intermediate' and 'dark' appear to be maintained by contrasting selection pressures on the extreme morphs, with darker individuals being less aggressive and more dispersive but more susceptible to ectoparasites, whereas light individuals are more aggressive, disperse later and carry more endoparasites [13–15]. Consequently, intermediately melanised birds tend to experience the best of both worlds and have the greatest overall lifetime reproductive success [13, 16].

Another potential mechanism by which melanin-based colouration may affect fitness involves differences in energy homeostasis [17–19]. Studies of several vertebrate species have shown that colouration can affect the regulation of food intake and energy expenditure, resulting in variation in adult body size [8, 18]. Typically, highly melanised individuals tend to be both larger and heavier than individuals with lighter colouration (reviewed by [8]).

Numerous songbird species are plumage polymorphic [20], making this group well suited to exploring the fitness consequences of plumage polymorphism. Within the Estrildid finches, for example, Gouldian finches possess different plumage morphs that are stable in the wild [21] and which differ in their fitness [22, 23]. The zebra finch (*Taeniopygia guttata*) provides another potential avian model system for exploring the effects of plumage colouration on morphological and fitness variation. This Estrildid finch has distinctive melanin based plumage ornaments that differ in the extent to which eumelanin and phaeomelanin are present [24]. Furthermore, variation in plumage colouration occurs naturally as well as extensively in domesticated populations in the form of different colour morphs, of which over 30 have been documented [25]. Although the colour morphs of parents and other conspecifics play a role in mate preferences and song development [26–28], little is known in general about the broader behavioural and fitness consequences of these plumage polymorphisms. Several studies have uncovered effects of colour morph on nest preferences [29] and it is also known that white zebra finches have a partially impaired visual system [30, 31], but no effects of morph have been found on cognitive skills [32].

Although zebra finches are in many respects ideally suited to exploring the fitness consequences of plumage polymorphism, they also exemplify an issue that is common to many association studies–that of the potentially confounding effects of cryptic population structure. It has long been recognised in studies of humans that associations between genotypes and phenotypes can be confounded by population stratification, which can lead to either false positives or false negatives [33]. Various approaches have been proposed to deal with this problem [34] but these typically rely on the use of very large panels of unlinked genetic markers, which are unavailable for most non-human study systems. Consequently, the majority of studies of colour polymorphisms in birds and other organisms have not been able to control for differences in the genetic backgrounds of individuals [8]. While it remains unclear how important this could be in wild populations, many of which do not appear to be strongly structured, population stratification may be more prevalent in domesticated populations, especially where multiple stocks are present.

**Fig 1:**
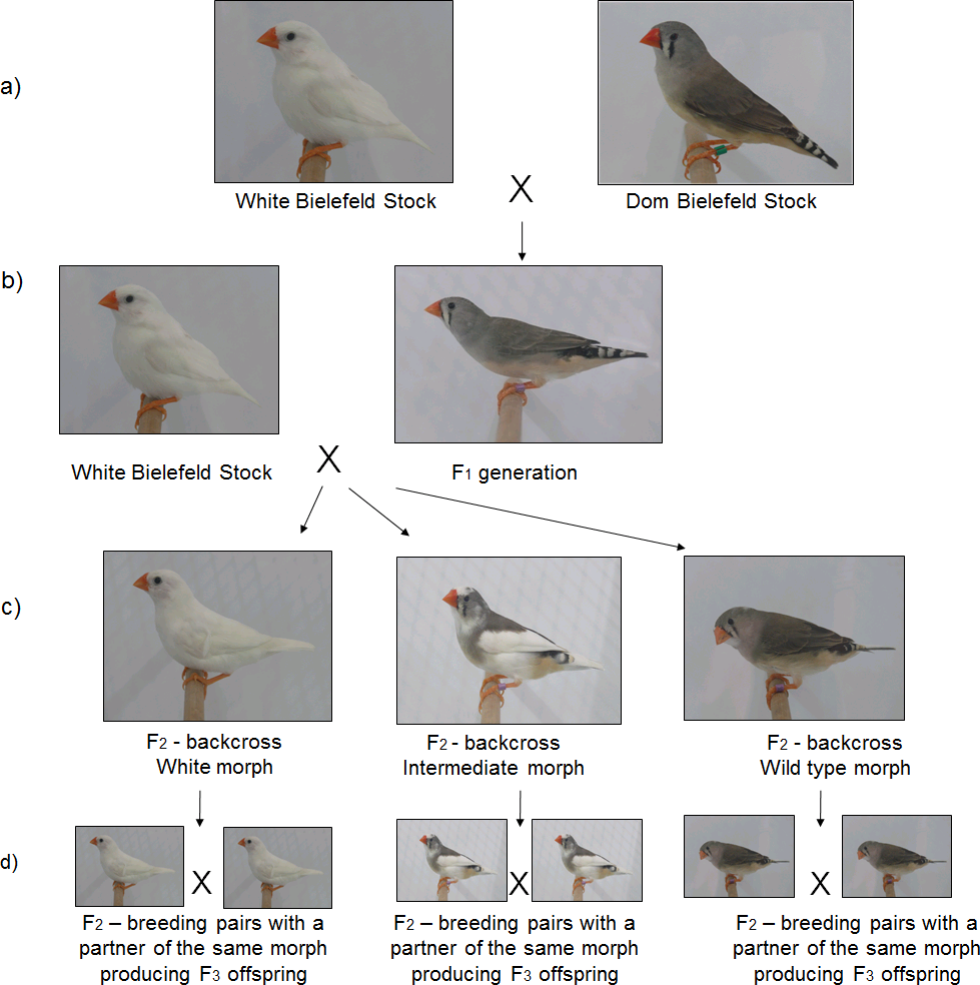
Overview of the controlled breeding experiment. a) F_0_ birds, b) F_1_ birds were backcrossed with white birds, resulting in c) F_2_ backcrossed birds, d) the F_2_ birds were paired with a partner of the same plumage morph. Photos are taken from [35, 66].

An example of this is provided by a recent study of zebra finches that looked for sequence differences in the *MC1R* gene between two plumage morphs that differ maximally with regard to the extent of melanin-based coloration, termed 'wild-type' and 'white' respectively [35]. Wild-type birds (Fig 1) are greyish in colour with males carrying additional secondary sexual ornamentation in the form of orange cheek patches, a black throat, black breast stripes and maroon-coloured flanks [25, 36]. By contrast, the white morph (Fig 1), which has an autosomal recessive mode of inheritance [25], is leucistic and completely lacks both phaeomelanin and eumelanin. Sequencing of the MC1R gene uncovered a highly significant association with morph, but this was confounded by strong underlying population structure as revealed by microsatellites. After crossing the two plumage morphs over two generations and rearing the F_2_ birds to adulthood (for details of the breeding scheme, see Fig 1), the original association between *MC1R* genotype and colouration was lost [35].

A further interesting observation is that the white zebra finch colour morph only occurs sporadically in the wild [25]. So far, it is unclear whether this is simply a reflection of low underlying allele frequencies, or whether white birds could be under stronger selective pressures than their wild-type counterparts. Overall, the consequences of plumage colour variation in zebra finches on physiology, behaviour and fitness have been rarely addressed in experimental studies, as pointed out by a recent review [37]. Consequently, in this study, we compared morphological and life-history traits between wild-type and white zebra finches using F_2_ birds from the experiment described above to control for background genetic differences. Specifically, we reared the F_2_ birds to adulthood, collected morphological measurements from them and then randomly paired birds of the same plumage morph and gave them the opportunity to breed. Given that the white morph is rare in the wild [25] and that darker individuals of many vertebrates are often in better body condition than lighter ones [8] we hypothesized that wild-type birds should have a selective advantage over white birds. Specifically, we expected to find that wild-type birds would be superior to white birds in key fitness-related traits such as body condition and reproductive investment.

## Methods

### Experimental animals

We used adult zebra finches belonging to the second generation (F_2_) of a breeding experiment that backcrossed individuals to obtain different plumage morphs while mixing the genetic background [35]. The breeding protocol is described in detail by Hoffman et al.[35] and is summarised in Fig 1. Briefly, to obtain the F_1_ generation, we first crossed domesticated zebra finches with wild-type plumage (N_F0 wild-type_ = 10) colouration (Population #19 in [38]; Dom Bielefeld, Fig 1) with zebra finches of the white plumage morph (N_F0 white_ = 10) (Population #20 in [38]; White Bielefeld, Fig 1). Individuals of the White Bielefeld plumage morph originated from a domesticated population that has been maintained in isolation since the 1990s. This population was initially founded using white individuals from the domesticated wild-type population (Dom-Bielefeld) as described in [29]. All but one of the F_1_ birds had wild-type plumage colouration, consistent with the white Bielefeld stock having previously been described as representing ‘a pure strain of an autosomal recessive leucistic mutant’ [35]. However, a single F_1_ bird had white plumage, suggestive of an unexpected mode of inheritance. Possible explanations include incomplete penetrance or the involvement of more than one locus [35].

To obtain the second generation, we then backcrossed the wild-type F_1_ birds (N_F1_ = 20) with white birds of the F_0_ population. All birds were assigned to one of the three plumage morphs, (i) wild-type; (ii) white; and (iii) intermediate. Intermediate birds had more than 25% of their total plumage assigned to both wild-type and white [35]. The result was a F_2_ generation with a total of 92 backcrossed birds comprising 22 with wild-type, 52 with white and 18 with intermediate plumage colouration (three additional birds with a fawn colouration were not used for the present study, as this morph has a sex-linked rather than autosomal mode of inheritance [25]). Of these, 77 survived until the start of the current study, comprising 21 birds with wild-type plumage colouration (9 ♀ and 12 ♂), 41 birds with white plumage colouration (21 ♀ and 20 ♂) and 15 birds with intermediate plumage colouration (6 ♀ and 9 ♂). Biometric measurements from these birds were collected at adulthood (average age = 391 days ± 56 S.D.). Body mass was recorded to the nearest 0.01g on a Sartorius PT120 electronic scale, wing length was measured to the nearest 0.5mm using a ruler, and tarsus length was measured to the nearest 0.1mm using digital callipers (Horex).

### Genetic analysis

All individuals were genotyped at eight polymorphic microsatellite loci as described in [35]. First, to confirm that the breeding experiment had eliminated the strong underlying pattern of population structure described in [35], we subjected microsatellite data for the 77 surviving F2 individuals to principle component analysis (PCA) within the R package [39]. The resulting PCA plot shown in Figure A in S1 File revealed no clear genetic differences between wild-type, intermediate and wild white individuals. Second, to control for any possible effects of heterozygosity on growth and reproduction, we calculated each individual's standardised multilocus heterozygosity (sMLH), a measure that corrects for the fact that not all individuals are genotyped for the same loci by standardizing the average multilocus heterozygosity of a focal individual by the average observed heterozygosities in the population of the subset of loci for which that individual was genotyped [40].

### Breeding experiment

We next allowed the birds to breed with partners of the same plumage morph (Fig 1) by forming 30 pairs, of which 9, 15 and 6 had wild-type, white and intermediate plumage respectively. Each pair was housed separately in an 83×30x40cm cage with an attached wooden-nest box (15x15x15cm) and coconut fibres were provided as nest material. Water baths were provided *ad libitum* and were refreshed three times per week [41]. High quality food (seed food supplemented daily with egg food and germinated seeds) was given to all breeding pairs *ad libitum* [42, 43]. Nest boxes were checked daily between 9 am and 12 am for the presence of new eggs and hatchlings. New eggs were marked using a non-toxic pen [44] and hatchlings were marked by trimming the down-feathers in an individually distinctive pattern [45]. Adult birds were allowed to initiate breeding for 60 days, at which point the experiment ended if no eggs were laid. All other experiments ended once the breeding pair had successfully raised a brood. For each pair, we measured the latency until the first egg was laid, the number of eggs laid, the masses of all eggs laid, and the number and weight of hatchlings. Only twelve out of 27 pairs that laid eggs had hatchlings, while the others 15 pairs that had laid eggs had zero nestling. This resulted in wild-type pairs having on average 1.87 hatchlings ± 1.64 (S.D.), intermediate pairs 1.0 hatchlings ± 2.45 (S.D.) and white pairs on average 1.46 hatchlings ± 1.85 (S.D.). From the pairs with hatchlings, the average number of fledging young was 2.4 ± 1.3 (S.D.) for wild-type birds and 2.0 ± 0.9 (S.D.) for white birds. Only one pair of intermediate birds had hatchlings, but five out of six of these young survived until fledging. The overall breeding success in our study was also only moderate with 40% of pairs producing hatchlings, which is lower than the mean of 64% in a study comparing several laboratories [37]. One explanation might be that the birds were breeding the first time.

### Statistical analysis

The following morphological traits were measured from all adult birds: body mass, tarsus length and body condition. The latter was calculated as the ratio of body mass to tarsus length and analysed using a Linear Mixed Effect Model (LME) with plumage morph fitted as a three level factor (wild-type / intermediate / white). Sex was fitted as a two level fixed factor (variable categories = male / female) and age at measurement and multilocus heterozygosity were also included as fixed factors (variable = numeric). The identity of the nest in which each adult was born was included as a random factor (the birds came from a total of 13 different nests). Where significant effects of plumage morph were found, *post hoc* analyses focused on pairwise comparisons of the relevant parameter estimates of the LME. To summarise effect sizes, we provided the R^2^values from the LME. The full model was reduced by excluding non-significant variables in a backward selection procedure to obtain the final model.

The following breeding parameters were quantified for all breeding pairs: (i) latency to laying the first egg; (ii) relative egg mass, calculated as mean egg mass / female body mass; (iii) the total number of eggs laid;(iv) total egg mass production effort, calculated as mean egg mass * the total number of eggs; and. (v) mean hatchling mass per brood. These variables were analysed using Linear Models (LMs) with only plumage morph fitted as a factor. Where significant results were obtained, *post hoc* analyses focused on pairwise comparisons using *post hoc* pairwise t-tests with pooled SD [46].

The latency to first egg was log transformed (log [X+1]) to obtain an approximately normal distribution the residuals of the model. However, a single outlier remained, which after removal resulted in the criteria of normality and homogeneity of variances being fulfilled. As the removal of this outlier did not appreciably influence the results of the model, we presented the model based on the full dataset in the results section and the model excluding the outlier in Section B in S1 File.

Model residuals of LMEs and LMs were tested for normality using Kolmogorow-Smirnow tests with Lilliefors correction, while homogeneity of variance was tested using Levene’s tests. For all models we provide the R^2^ as a measure of the total explained variance [47]. Associated *P*-values were provided without correction for multiple testing as our study had a relatively small number of planned tests [48, 49]. All tests were implemented within R 3.3.1 using the packages nlme [50], nortest [51] and lsr [46]. *Post hoc* power analyses for non-significant linear models were implemented using G*Power 3.1.9.2 [52]. The raw data from the experiments are available in S2 File.

### Ethical note

Housing and breeding of the zebra finches was carried out with the approval of the Veterinäramt Bielefeld (permit numbers 530.42 1630–1; 18.04.2002 and 530.4; 24.07.2014). All birds had always *ad libitum* access to food and water. Birds were visually monitored daily.

## Results

### Body condition variation among the plumage morphs

**Fig 2:**
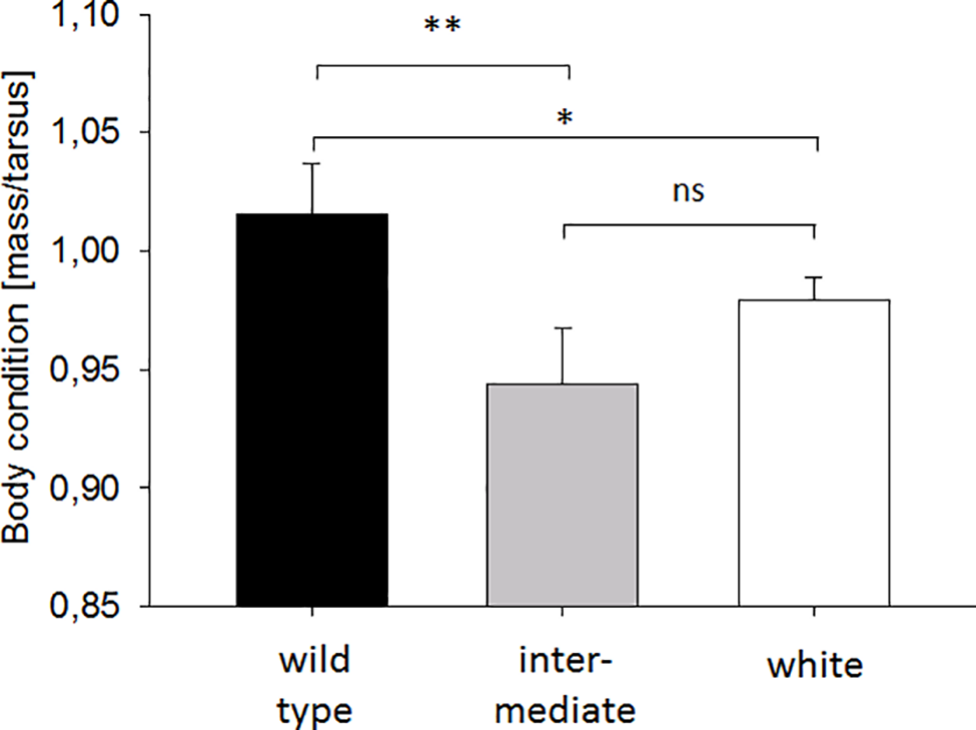
Body conditions of the three plumage morphs of the F_2_ birds prior to breeding. Shown are the means ± SE of each plumage morph. The three groups differ significantly from each other and the *post hoc* comparisons reveal differences between wild-type and intermediate birds (p = 0.002, indicated by “**”) and wild-type and white birds (p = 0.012, indicated by “*”). White and intermediated coloured birds did not differ from each other in body condition (p = 0.16, indicated by “ns”). See the texts for details of the tests.

Body condition, expressed as the ratio of body mass to tarsus length, differed significantly among the three plumage morphs and was also significantly higher in females relative to males (Fig 2; LME_condition_: factor plumage morph F_2,61_ = 7.66, p = 0.001, factor sex F_1,61_ = 20.06, p < 0.0001, R^2^ of the model = 0.63). *Post hoc* pairwise comparisons revealed significant differences between white and wild-type birds (p = 0.012) as well as between wild-type and intermediate birds (p = 0.002), whereas white and intermediate birds were not significantly different (p = 0.16) from one another.

### Effects of plumage morph on reproductive traits

**Fig 3:**
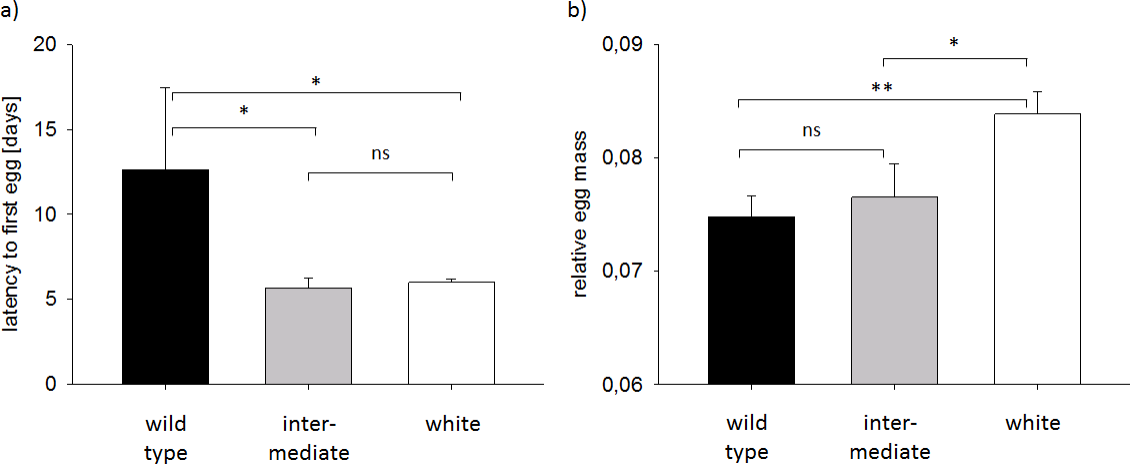
Reproductive parameters of the F_2_ breeding pairs belonging to one of the three plumage morphs. Shown are means ± SE for a) latency until pairs laid the first egg (in days); and b) relative egg mass (calculated as mean egg mass / female body mass). Latency until pairs laid the first egg was significantly different between the three plumage morphs and the *post hoc* comparisons revealed that wild-type pairs had higher latencies than both white and intermediate birds (both p<0.024, as indicated by “*”). Whereas latencies of intermediate and white pairs did not differ (p = 0.71, indicated by “ns”). The relative egg mass also differed between the three morphs and the *post hoc* test indicated that white birds had a higher relative egg mass than intermediate (p = 0.03, indicated by “*”) and wild-type birds (p = 0.005, indicated by “**”). Intermediate and wild-type birds did not differ (p = 0.63, indicated by “ns”). See text for details.

The majority of breeding pairs (27 out of 30) laid at least one egg during the experiment. Latency to lay the first egg varied significantly among the three plumage morphs (LM_latency to first egg_: factor morph F_2,24_ = 3.98, p = 0.03, R^2^ = 0.25; Fig 3A). *Post hoc* comparisons indicated that white and intermediate birds started laying eggs around the same time (p = 0.71; Fig 3A) and that both of these morphs began laying significantly earlier than wild-type birds (both p<0.024; Fig 3A).

The total number of eggs produced did not differ significantly by morph (LM_number of eggs_: factor plumage morph F_2,27_ = 2.04, p = 0.15, R^2^ = 0.13, Power = 0.37). However, female investment in terms of mean relative egg mass varied significantly (Fig 3B; LM_mean rel. egg mass_: factor plumage morph F_2,24_ = 5.55, p = 0.01, R^2^ = 0.32; Fig 3B). *Post hoc* comparisons showed that white pairs had higher values than intermediate (p = 0.03; Fig 3B) and wild-type (p = 0.005; Fig 3B) pairs but the relative egg masses of intermediate and wild-type birds did not differ significantly (p = 0.63; Fig 3B). The total effort in egg mass production did not vary significantly by plumage morph (LM_total effort in egg mass_: factor plumage morph F_2,24_ = 2.13, p = 0.14, R^2^ = 0.15, Power = 0.43). Similarly, plumage morph was unrelated to hatchling mass (LM_mean hatchling mass_: plumage morph F_2,8_ = 0.16, p = 0.86, R^2^ = 0.04, Power = 0.08).

## Discussion

Using zebra finches of different plumage morphs from a controlled breeding experiment, we showed that plumage colouration is associated with differences in adult body condition and reproductive traits. Birds of the white morph exhibited shorter latencies to start reproduction and tended to invest more into their eggs than wild-type birds after controlling for maternal body mass. By contrast, the morphs did not differ significantly with respect to either the total number or biomass of offspring, although our experiment had limited statistical power in respect of these particular traits. Taken at face value, our findings suggest that wild-type and white birds may be exploiting different reproductive strategies that under controlled laboratory conditions do not appear to result in a net difference in reproductive fitness. However, larger sample sizes in the laboratory as well as confirmatory work in the wild would be required to reach more confident conclusions in respect of the ultimate fitness outcomes.

Our study was partly motivated by the observation that, although both wild-type and white zebra finches are present in the wild, white animals are quite rare [36], although they have been maintained in captive populations through selective breeding [35, 38]. While frequency differences in the wild could simply be a reflection of the underlying allele frequencies, in which case the white allele should be relatively rare, another possibility is that white birds might be subjected to stronger natural selection under natural conditions. For example, highly conspicuous phenotypes like bright white colouration might be associated with increased predation pressure in the wild, as has been observed in several other species [53–55]. Alternatively, as shown for example in guppies, rare phenotypes could potentially accrue fitness advantages depending on the prevailing population structure [56]. This has been advocated in predators as the apostatic selection hypothesis [57] where a rare morph might not be as readily recognised as either a predator or prey due to its rarity, resulting in negative frequency-dependent selection.

In a previous study, we showed that laboratory strains of wild-type and white zebra finches differ significantly from one another in their genetic background as measured using microsatellites, which reflect their long history of selective breeding in isolation [35]. As population structure can be a problem for association studies [33, 34, 58, 59], we therefore used a controlled breeding design to reduce the risk of obtaining artefactual associations that reflect differences in the genetic background rather than the trait in question (i.e. plumage colouration). Having done so, we found a number of significant associations between plumage colouration and morphological and life-history traits. It remains unclear whether these associations could be directly caused by the gene(s) responsible for colouration, or whether genes in linkage disequilibrium with them could be involved. Direct effects are plausible as *POMC*, for example, is expressed in multiple tissues and can have downstream influences on diverse processes ranging from stress responsiveness, food intake, energy expenditure and sexual activity [8]. However, given the unusual recombination landscape of zebra finches, in which almost a third of the genome typically segregates in only four independent blocks [60], possible effects of linked genes cannot be discounted.

One of our main findings was that wild-type and white birds differed significantly in body condition. White birds on average had lower body condition in adulthood than darker birds, consistent with findings in other vertebrate species [8]. However, white zebra finches appeared to compensate for these differences during reproduction by laying earlier and investing a significantly greater proportion of their total body mass into eggs. This increased investment would be expected to be beneficial in the short term, as egg mass correlates positively with hatchling mass in zebra finches, and heavier chicks in turn have a greater likelihood of surviving into adulthood [43]. Compensation in reproductive traits has been shown in other contexts in zebra finches. For example, females paired with an attractive partner invest more into their eggs [61], whereas breeding pairs that have experienced harsh conditions during early life tend to have a shorter latency to reproduce [62]. Flexibility in these traits would thus appear to be adaptive, at least in the short term, although compensatory investment may well carry costs later in life as one would expect there to be a trade-off between reproductive and somatic investment [63–65].

The potential costs arising from compensation in important fitness-related traits are worthy of further study. We focused on birds during their first breeding attempt, which seems reasonable as zebra finches are opportunistic breeders [25] and may not always have multiple opportunities to reproduce. However, as studies of other bird species have uncovered links between lifetime breeding success and plumage colouration [16], an obvious next step would be to investigate the effects of colour morph over the entire lifespan in order to test whether the patterns we have uncovered remain stable throughout adulthood or whether they diminish over time. Such a study would also be long enough to test for differences between the morphs in rates of senescence and longevity.

During our experiment, compensatory investment on the part of white zebra finches appeared to result in similar levels of overall reproductive investment as for wild-type birds, despite the latter being in better body condition. If this is indeed the case, our findings would contrast with several studies of wild vertebrate populations (e.g. owls, buzzards or Gouldian finches [9–11, 13, 23]) showing that colour morphs often differ markedly in respect of key fitness traits and ultimately reproductive success. We can think of two main possible explanations for such a discrepancy. First, the results of *post hoc* power tests corresponding to our non-significant statistical analyses revealed generally rather limited power, particularly for the effect of plumage morph on hatchling mass. This suggests that some of our tests lacked the statistical power to detect anything but very strong effects, an issue that could potentially be remedied in future studies by further increasing sample sizes. Second, our experiment was carried out under controlled laboratory conditions where high quality food was provided *ad libitum* and the birds were not exposed to predators. Under more natural conditions, any fitness differentials should be amplified, a possibility that could be tested by replicating our study in semi-natural enclosures and / or by manipulating food availability. Finally, there is also the question of how representative our study may be of the situation in the wild, as our crossing design will have altered the genetic background of the colour polymorphism(s) responsible for the white phenotype. To evaluate this, a confirmatory study would be required in the wild, although this may prove difficult due to the natural rarity of white birds.

To summarise, our study uncovered a number of differences between wild-type and white zebra finches after having controlled for the potentially confounding effects of population structure through a controlled breeding design. Specifically, white birds were significantly lighter and had lower body condition than wild-type birds, yet they reproduced earlier and laid heavier eggs relative to their own body mass. The net result was no significant difference in total reproductive output, although our study was underpowered to detect anything but very large differences in total egg mass production and total hatchling mass. Although it appears that differences in morphological traits between wild-type and white birds might be compensated for during reproduction in captivity, it may be more difficult for white birds to compensate for their lower body condition under natural conditions, which could potentially help to explain the low frequency of white birds in the wild. Furthermore, due to their conspicuous phenotype, white birds may also suffer from greater predation under natural conditions.

## Supporting information

S1 File Contains supplementary figure and supplementary information. Fig A. Results of the principle component analysis of microsatellite data from 77 surviving F_2_ individuals (see Materials and methods for details). The x and y axes represent the first and second principal components respectively. Individuals are represented by dots and the colour morphs are colour coded (wild-type = dark brown, intermediate = light brown and white = cream) and depicted by 95% inertia ellipses. Section B). Output of the model excluding the single outlier for the latency to lay the first egg.
(DOCX)

S2 File Contains the raw data. Raw data from the experiment are presented in these tables.
(XLSX)
