# Supplementary material for: The influence of inherited plumage colour morph on morphometric traits and breeding investment in zebra finches (Taeniopygia guttata)
Source: PLoS One. 2017 Nov 30;12(11):e0188582. doi: 10.1371/journal.pone.0188582 (PMC5708660; doi:10.1371/journal.pone.0188582)
Supplement: S1 File — Fig A. Results of the principle component analysis of microsatellite data from 77 surviving F2 individuals (see Materials and methods for details). The x and y axes represent the first and second principal components respectively. Individuals are represented by dots and the colour morphs are colour coded (wild-type = dark brown, intermediate = light brown and white = cream) and depicted by 95% inertia ellipses. Section B). Output of the model excluding the single outlier for the latency to lay the first egg. (DOCX) [file pone.0188582.s001.docx]

# Supplementary material – File S1

# **The influence of inherited plumage colour morph on morphometric traits and breeding investment in zebra finches (*Taeniopygia guttata*)**

E. Tobias Krause^1,2,*^, Oliver Krüger^1,^, Joseph I. Hoffman^1,*^

^1^ Department of Animal Behaviour, Bielefeld University, Morgenbreede 45, 33615 Bielefeld, Germany

^2^ Institute of Animal Welfare and Animal Husbandry, Friedrich-Loeffler-Institut, Dörnbergstr. 25-27, 29223 Celle, Germany

**Supplementary Figure A**


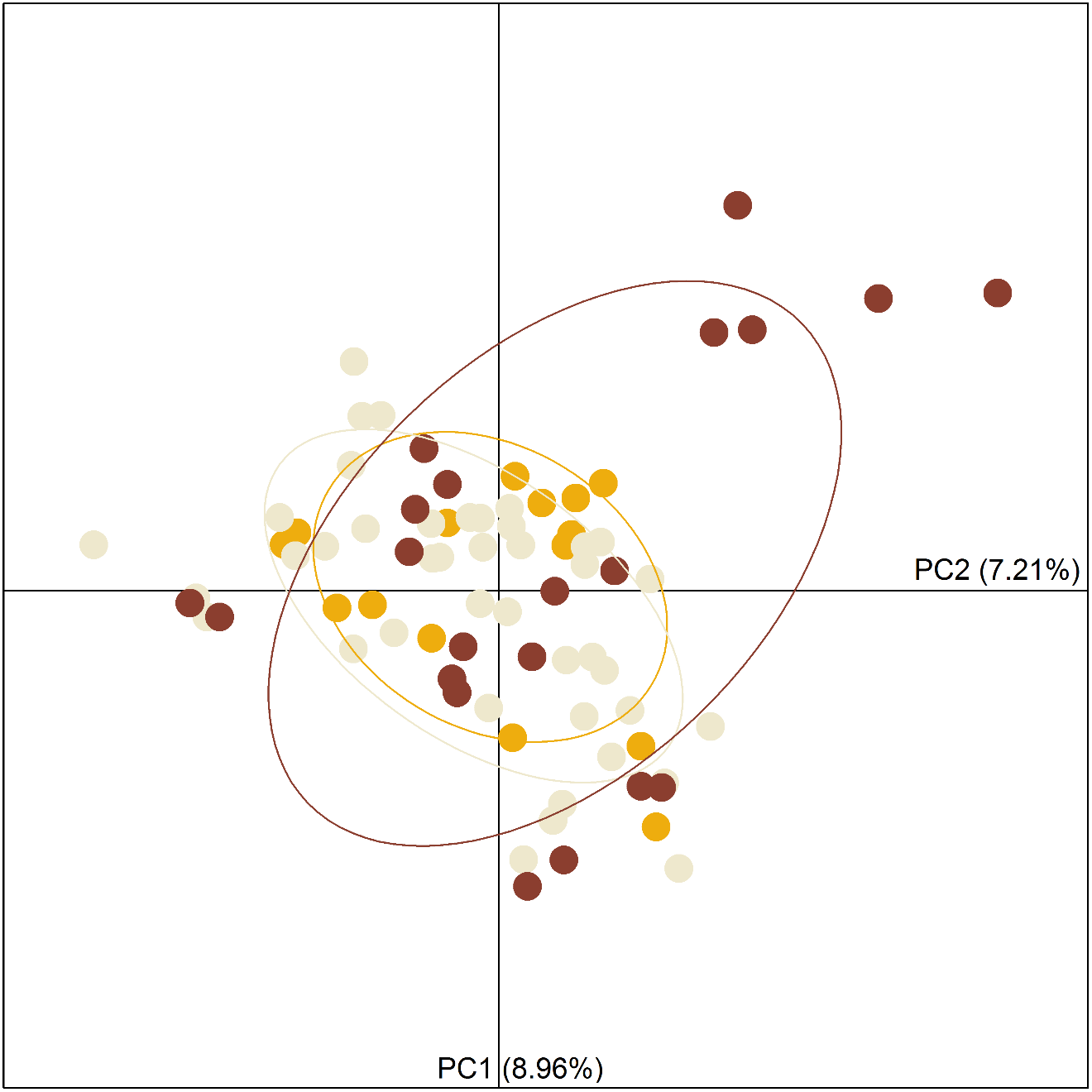


**Fig. A:** Results of the principle component analysis of microsatellite data from 77 surviving F2 individuals (see Materials and methods for details). The x and y axes represent the first and second principal components respectively. Individuals are represented by dots and the colour morphs are colour coded (wild-type = dark brown, intermediate = light brown and white = cream) and depicted by 95% inertia ellipses.

**Section B:** **Output of the model excluding the single outlier for the latency to lay the first egg**

The latency to lay the first egg remained significantly different among the pairs of the three morphs after excluding a single outlier (LM_latency to first egg_: factor morph F_2,23_ = 3.85, p = 0.036).
